# Low-cost biosurfactant production by *Achromobacter xylosoxidans* PX106473 from waste frying oil: partial characterization and antimicrobial mechanism via molecular docking

**DOI:** 10.1186/s12934-025-02906-7

**Published:** 2026-01-10

**Authors:** Sally M. Elmogy, Magda M. Awad, A. M. M. Elattaapy, El Sayed F. El-Halawany, Ashraf A. Elsayed

**Affiliations:** 1 Botany Department, Faculty of Science, Mansoura University, Elgomhouria St., 35516 Mansoura, Egypt; 2 Microbiology Department, Faculty of Agriculture, Mansoura University, Elgomhouria St., 35516 Mansoura, Egypt

**Keywords:** Biosurfactant, Achromobacter xylosoxidans, Lipopeptides, Characterization, Waste frying oil, Molecular docking.

## Abstract

**Background:**

Interest in microbial biosurfactants has increased due to the rising demand for environmentally friendly and sustainable surfactants. Waste frying oil provides a renewable and low-cost feedstock for their production. This study aimed to isolate, characterize, and evaluate the antibacterial mechanism of a biosurfactant synthesized by *Achromobacter xylosoxidans* PX106473 using waste frying oil as an economical carbon source.

**Results:**

*Achromobacter xylosoxidan*s PX106473 produced a biosurfactant with significant activity, including an emulsification index (E24%) of 66.7% against kerosene and substantial oil displacement and hemolytic activities. According to the results of thin-layer chromatography (TLC), the produced biosurfactant contained lipids and amino acids. Fourier transform infrared spectroscopy (FT-IR) results revealed the presence of an N-H group, aliphatic hydrocarbons, and amide peaks, which suggest a lipid-peptide linkage, providing further evidence for its putative lipopeptide nature. Hexadecanoic acid, with an area percentage of 76.44, was the dominant component of the lipopeptide based on gas chromatography-mass spectrometry (GC-MS) results. The produced biosurfactant demonstrated good inhibitory activity against *E. coli* and *S. aureus*. These biological findings were further supported by in silico assays; molecular docking studies showed that hexadecanoic acid binds stably to key bacterial proteins from *E. coli* (DNA gyrase B, -6.4 kcal/mol) and *S. aureus* (PBP2a, -3.9 kcal/mol), indicating a potential dual-target mechanism.

**Conclusion:**

*Achromobacter xylosoxidans* efficiently produced a putative lipopeptide biosurfactant from waste frying oil with strong emulsifying and antibacterial properties, providing an economical and sustainable solution with potential in various environmental and pharmaceutical applications.

## Background

The demand for surfactant production has expanded recently due to its widespread applications in several industries. The global surfactant market, valued at $41.3 billion in 2019 [1], is forecasted to grow, reaching $58.5 billion by 2027 [2]. Despite the high global demand for surfactants, the production of chemical surfactants has accelerated the depletion of natural resources and climate change, representing a serious environmental concern [3]. Hence, it is essential to produce surfactants that are more sustainable and environmentally friendly [4].

Biosurfactants, or microbial surfactants, are defined as a unique class of structurally diverse amphiphilic compounds that can lower the surface tension of liquid interfaces. They are produced by various microbes, such as bacteria, fungi, and yeast [5]. The interest in biosurfactants has increased over the past few years, mainly because of their eco-friendly characteristics and the possibility of production using waste materials [6]. Additionally, producing biosurfactants from renewable substrates may help overcome the problem of high production cost resulting from recovery and purification techniques [7]. Agro-industrial and food processing residues, including soybean molasses, starchy byproducts, animal fats, and used frying oil, have shown potential as alternative carbon and nitrogen sources for fermentative biosurfactant production [8]. Oily waste materials such as waste frying oil, known to contribute to serious environmental issues, proved to be effective, cheap, and renewable carbon sources for biosurfactant production [9, 10]. Domestic waste cooking oil also contains a balanced nutrient composition that supports optimum bacterial growth and enhances biosurfactant synthesis [11].

Biosurfactants offer several advantages over synthetic chemical surfactants, including good environmental affinity, high selectivity, higher biodegradability, higher foaming, low toxicity, multi-functionality, availability of resources, and stability at higher temperatures and extreme pH [12]. As such, these ‘wonder molecules’ have numerous applications, including detergents, emulsifiers, foaming agents, wetting agents, and the formulation of food additives, household cleaners, paints, healthcare products, and printing materials [13]. In addition, biosurfactants are widely employed in various environmental and biomedical applications, such as oil spill remediation [14], anti-cancer agents, antimicrobials for cosmetics industry, and anti-adhesive agents against various bacterial and yeast pathogens for medicinal purposes [15]. Biosurfactants are also known for their inhibition activity against *Bacillus cereus*, *Escherichia coli*,* Vibrio cholera*, and *Klebsiella pneumonia* [16].

Biosurfactants are broadly categorized by molecular weight. Low molecular weight biosurfactants, such as glycolipids and lipopeptides, effectively reduce surface tension. High molecular weight bioemulsifiers, including lipopolysaccharides and proteins, primarily stabilize emulsions [17]. Glycolipids have many applications, for example, petroleum bioremediation, enhanced oil recovery, and heavy metals removal from soil [18]. Lipopeptides are also valued for their antimicrobial, antitumor, and immunosuppressive functions and are generally more effective than glycolipids in lowering water surface tension [19, 20].

So, our study aimed to isolate and characterize a biosurfactant produced by *A. xylosoxidans* PX106473 using waste frying oil as a renewable feedstock and a cheap source of carbon. The produced biosurfactant was further evaluated for its antibacterial activity against *E. coli* and *S. aureus.* Additionally, molecular docking studies were conducted to understand the antibacterial mechanism of the isolated biosurfactant and to investigate its interaction with the surface proteins of *E. coli* and *S. aureus*.

Although several microbial genera have been investigated for biosurfactant production, no study has characterized a lipopeptide-type biosurfactant from the *Achromobacter xylosoxidans* PX106473 strain using waste frying oil. Furthermore, this study uniquely combines experimental characterization with molecular docking analysis to elucidate the antibacterial mechanism of the dominant biosurfactant component (hexadecanoic acid). This integrated experimental-computational approach provides new insight into the antimicrobial mechanism of lipopeptide biosurfactants from *A. xylosoxidans* PX106473 that have not been reported in earlier *Achromobacter*-related biosurfactant studies.

## Materials and methods

### Bacterial strain and chemicals

*Strain* Previously isolated *Achromobacter xylosoxidans* PX106473 originally obtained from the bacteriology laboratory, botany department, Mansoura University, Egypt.

*Chemicals* The Bushnell Hass medium (BHM) used for the isolation and characterization of biosurfactant had the following composition; of 1 g KH_2_PO_4_, 1 g of NH_4_NO_3_.7H_2_O, 1 g K_2_HPO_4_, 0.02 g CaCl_2_, 0.2 g MgSO_4_, and 0.002 g of FeCl_2_ in a total volume of 1,000 mL enriched with 2% (v/v) waste frying oil [21]. Waste frying oil, as the only carbon source for biosurfactant production, was filtered using a filter paper and stored at room temperature.

### Detection of biosurfactant production by *A. xylosoxidans* PX106473

For the detection of biosurfactant production by *A. xylosoxidans* PX106473, the seed culture was inoculated into one flask containing a 100 ml fermentation medium, incubated at 30 °C for 7 days under shaking at 150 rpm, and the initial pH of the medium was adjusted to 7.0 before sterilization. After the fermentation process, the crude yield of biosurfactant, the emulsification index test (E24%), oil displacement assay, and haemolytic activity were detected.

The culture was.

#### **Emulsification index test (E24%)**

*A. xylosoxidans* PX106473 was cultured for one week to calculate the E24%. At first, 100 mL of BHM was centrifuged for 10 min at 4 °C and 6,000 rpm, and the supernatant was collected. Then, equal volumes of the supernatant and kerosene were added. The emulsion was vigorously mixed using a vortex and fixed for 24 h in a place. The uninoculated fermentation medium was used as a negative control and consistently showed no emulsification. The height of the resulting emulsion layer was recorded. The assay was performed with three replicates (*n* = 3), and the emulsion index (E24%) was calculated using the following formula;

$$ {\mathrm{E24}}\% \, = \,\left( {{{h~emulsion} \mathord{\left/ {\vphantom {{h~emulsion} {h~total}}} \right. \kern-\nulldelimiterspace} {h~total}}} \right) \times 100 $$

where h emulsion is the emulsion layer height, h total is the total liquid height, and E24% is the emulsion index after 24 h [22, 23].

#### Oil displacement test

The oil spreading technique was carried out following the methodology reported by [24, 25]. 40 mL of distilled water was poured into a petri dish, and 50 µL of waste oil was applied to the water surface. Then, 5 µL of the cell-free supernatant from a 7-day-old *A. xylosoxidans* PX106473 culture was applied. The diameter of the clear zone formed on the oil surface was measured. The activity of a surfactant is indicated by the size of the oil-displaced circle it creates.

#### Hemolytic activity

The hemolytic activity of *A. xylosoxidans* PX106473 was assessed by blood agar supplemented with 5% (v/v) fresh blood. The inoculated plate was incubated for 48 h at 37 °C. The formation of a clear zone surrounding the bacterial colonies indicated hemolysis and was interpreted as evidence of biosurfactant production [26].

### Extraction of biosurfactant from *A. xylosoxidans* PX106473 using waste frying oil

*A. xylosoxidans* PX106473 strain was tested for the biosurfactant extraction. Initially, the BHM was centrifuged for 30 min at 5 °C and 5000 rpm. The supernatant was collected, and its pH was adjusted to 2.0 with HCl (6.0 M). The acidified supernatant was kept at 4 °C for 24 h. Following this, an equivalent volume of a mixture containing chloroform-methanol (2:1, v/v) was added, vigorously mixed, and incubated overnight at 25 °C. The interfacial layer, which contained biosurfactant, was subsequently separated and subjected to another round of centrifugation for 30 min at 5 °C and 5000 rpm. The resulting pellet was identified as the crude biosurfactant. A sterile petri dish was weighed, the crude biosurfactant was transferred to it, and the combined weight was recorded. The dish was put in a hot air oven for 30 min at 100 °C to allow for drying. The biosurfactant yield was measured as dry weight, expressed in (µg/ mL). The dried biosurfactant extract was subsequently analyzed to determine its chemical nature.

### Characterization of the extracted biosurfactant

#### Thin-layer chromatography (TLC)

The extracted biosurfactant/methanol solution was applied onto a silica gel chromatography plate as a stationary phase. Separation of the components was achieved using a mobile phase consisting of chloroform and methanol in an 80:20 (v/v) ratio. After drying, the chromatographic plate was treated with three different chromogenic reagents to detect specific functional groups: ninhydrin solution (0.5% ninhydrin in acetone) to detect amino acids, iodine vapors to detect lipids, and phenol-sulphuric acid solution (3% phenol and 5% sulphuric acid in ethanol) to detect carbohydrates [27].

#### Fourier transform infrared (FT-IR) spectroscopy

One mg of the extracted biosurfactant was finely ground with 100 mg of potassium bromide (KBr) and compressed for 30 s under 7500 kg in order to form a translucent pellet. Using a JASCO FT-IR 4100 spectrometer, infrared spectra were recorded over a wavenumber range of 4000–400 cm⁻¹. Each measurement was conducted with 500 scans, using a KBr pellet as the background reference. FT-IR result was analyzed by examining the stretching vibrations of the functional groups present in the extracted biosurfactant sample [28].

#### Gas chromatography-mass spectrometry (GC-MS)

The extracted biosurfactant was subjected to high-resolution GC-MS analysis following the method described by [28]. 10 mg of the biosurfactant was mixed with 5% HCl in 1 mL methanol. 1 mL of water was added to quench the reaction. The sample was analyzed using a Trace GC-TSQ mass spectrometer (ThermoScientific, Austin, TX, USA), equipped with a capillary column and a mass-selective detector that scans at a rate of 1.2 scans per second between m/z 45 and m/z 800. The oven temperature was set to increase by 2 °C every minute from 130 °C to 230 °C. Helium was the carrier gas at a flow rate of 1 mL per minute with a split ratio of 50:1. The mass spectra of the fatty acids and fatty acid methyl esters present in the extracted biosurfactant were analyzed using the National Institute of Standards and Technology spectral libraries and WILEY 09 [29].

### Antibacterial activity of the extracted biosurfactant

The extracted biosurfactant’s antibacterial activity was evaluated against *E. coli* and *S. aureus*, procured from our bacteriology lab at the Faculty of Science, Mansoura University, Egypt, using the well diffusion assay [30]. Briefly, overnight-cultured strains were inoculated into LB medium separately. Purified biosurfactant was dispensed into wells of these nutrient agar plates that had been pre-seeded with the respective bacterial inoculum. The plates were then incubated for 24 h at 37 °C. The assay was conducted with three independent replicates (*n* = 3) for each bacterial strain, and the data are presented as the mean zone of inhibition ± standard deviation [31].

### Computational studies

#### Ligand preparation

Hexadecanoic acid was selected based on GC-MS analysis. The 2D chemical structures of the compounds were drawn using MarvinSketch, an advanced molecule editor from ChemAxon (MarvinSketch, ChemAxon, version 22.17.0) (available at https://chemaxon.com). This structure was then energy-minimized and converted to a 2D conformer in mol2 format, followed by conversion to Protein Data Bank (PDB) format, which was used for docking [32].

#### Protein preparation

Two bacterial protein targets, identified by their Protein Data Bank (PDB) IDs, were downloaded from the RCSB PDB database [33]: 1VQQ (penicillin-binding protein mecA from *Staphylococcus aureus*) and 5MMN (DNA gyrase subunit B from *Escherichia coli*). Water molecules, heteroatoms, and co-crystallized ligands were removed using PyMOL Molecular Graphics System (version 2.5.4) [34] to retain the native protein structure only. The cleaned PDB structures were saved for docking.

#### Molecular docking

Docking simulations were carried out using PyRx 0.8, which integrates AutoDock Vina for virtual screening [35]. The ligand was imported into PyRx and converted to PDBQT format using OpenBabel, which is embedded within the software. The proteins were also converted into PDBQT format after preparation. Grid box parameters were optimized to cover the entire active site of the target protein. AutoDock Vina was used with default settings to dock hexadecanoic acid to both target proteins. The binding affinity (kcal/mol) was recorded for all docked complexes.

#### Visualization and interaction analysis

The docking poses and ligand-protein interactions were visualized using PyMOL [34]. The hydrogen bonds, binding residues, and 3D orientation of ligands within the protein active sites were analyzed and recorded. The most stable conformations with the lowest binding energy were selected for further discussion.

## Results

### Detection of biosurfactant production by *A. xylosoxidans* PX106473

**Fig. 1:**
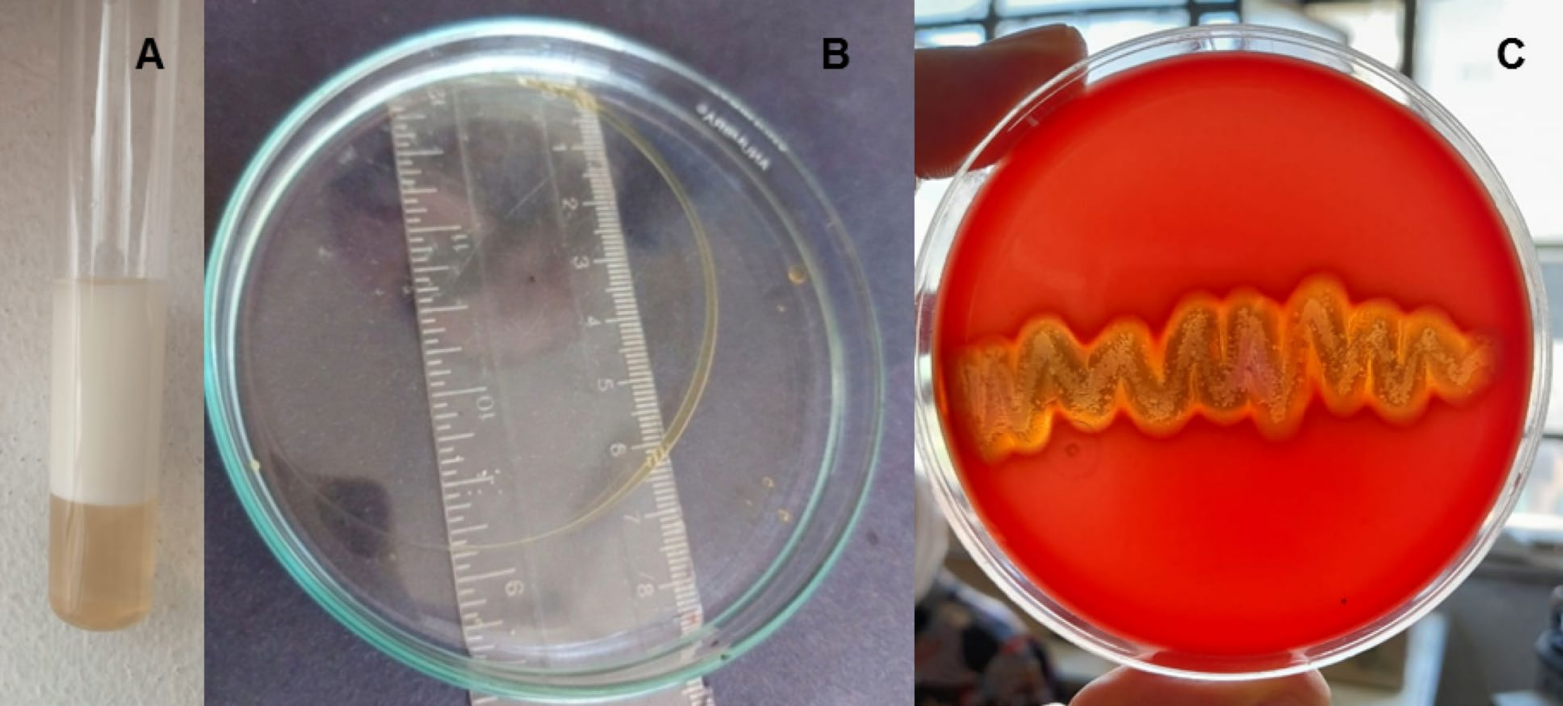
Emulsification index (E_24_) of *Achromobacter xylosoxidans* PX106473 **A**. Oil spreading activity by *Achromobacter xylosoxidans* PX106473 **B**. Colonies of *Achromobacter xylosoxidans* PX106473 formed on a blood nutrient agar plate **C**

The ability of *A. xylosoxidans* PX106473 to produce biosurfactants was confirmed through its emulsification activity against kerosene. A high emulsification index was observed with 66.7 ± 0.14% activity (Fig. 1A). The oil spreading test is a quick way for evaluating a surfactant’s surface activity; a larger clear zone diameter reflects greater activity of the tested biosurfactant. The result of the oil spreading activity by *A. xylosoxidans* PX106473 showed a large, clear zone with a 7 cm diameter (Fig. 1B). Biosurfactant production by *A. xylosoxidans* PX106473 was preliminarily assessed through haemolysis screening, where the appearance of a clear zone surrounding the colonies indicated positive activity of the biosurfactant produced by *A. xylosoxidans* PX106473, as shown in Fig. 1C.

### Characterization of the produced biosurfactant with TLC

The biosurfactant was purified at a crude level to enable structural screening with a concentration of 190 µg/mL based on dry weight measurement. TLC analysis of the produced biosurfactant by *A. xylosoxidans* PX106473 was performed and visualized using three chromogenic reagents. The silica gel plate showed yellow and red spots with iodine vapors and ninhydrin, indicating positive reactions of both lipids and amino acids, respectively. In contrast, no color was observed with the phenol-sulphuric acid reagent, suggesting the absence of carbohydrate components in the tested biosurfactant.

### Biosurfactant analysis by (FT-IR) spectroscopy

**Fig. 2:**
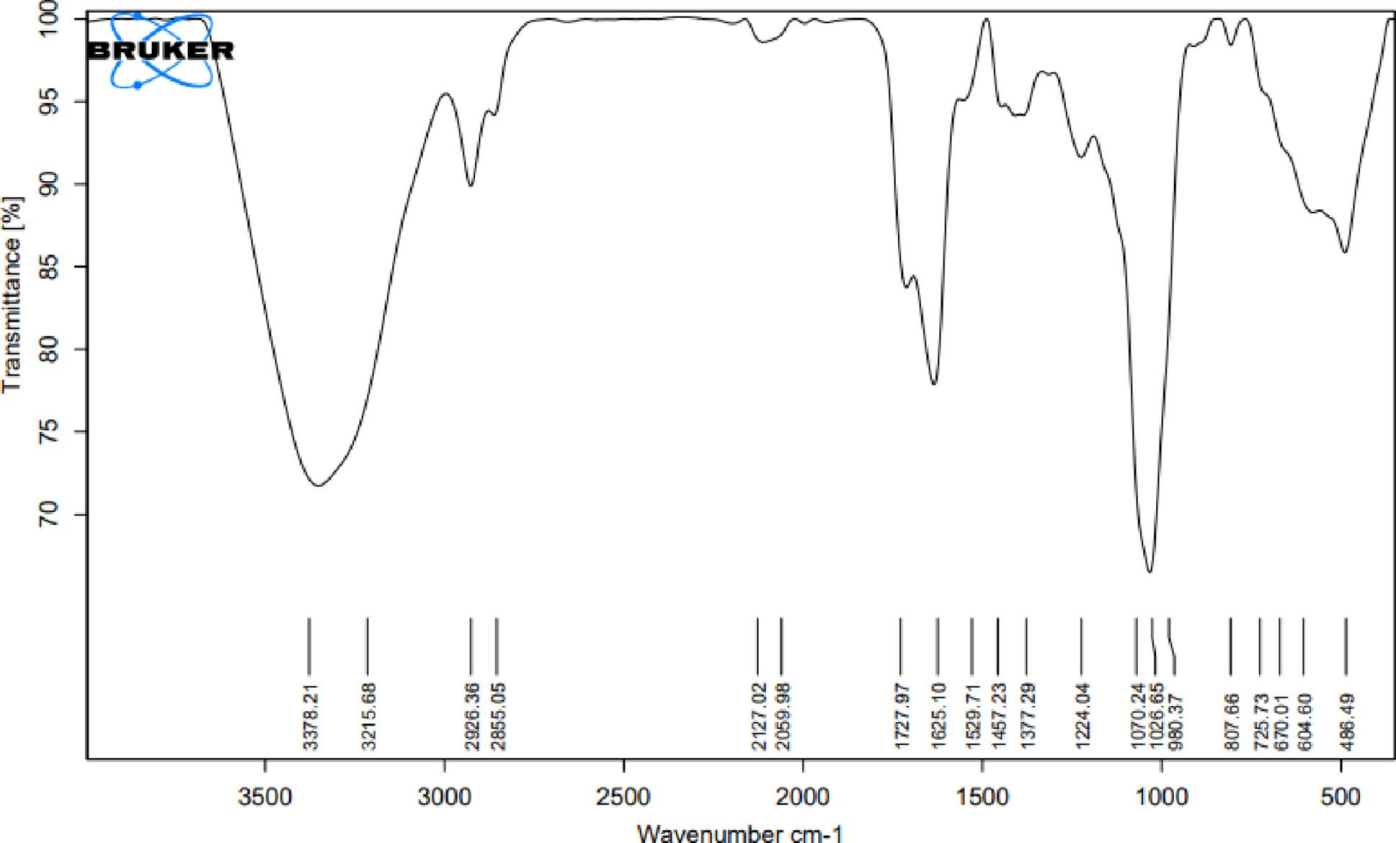
FT-IR spectrum of the crude biosurfactant produced by *A. xylosoxidans* PX106473

Infrared spectroscopy was used to chemically characterize and identify the biosurfactant produced by *A. xylosoxidans* PX106473 (Fig. 2). The absorbance values include 3378 cm^–1^ and 3215 cm^–1^ broad bands, corresponding to (O-H) and (N-H) stretches, respectively. The stretching bands around 2926 cm^–1^ and 2855 cm^–1^ are from the C-H stretching vibrations. The significant absorption band observed at 1727 cm⁻¹ was from the carbonyl (C = O) functional group. The sharp band at 1625 cm^− 1^ corresponded to the CO-N bond, and 1529 cm^− 1^ indicated the stretching mode of the C-N bond. 1457 cm⁻¹ and 1377 cm⁻¹ peaks are commonly related to symmetric CH_2_ and CH_3_ bending, respectively. C-O stretching vibrations had strong bands at 1070 cm⁻¹ and 1026 cm⁻¹, which are characteristic of carboxylic acids and their derivatives.

### GC-MS of fatty acids in the extracted biosurfactant

**Fig. 3:**
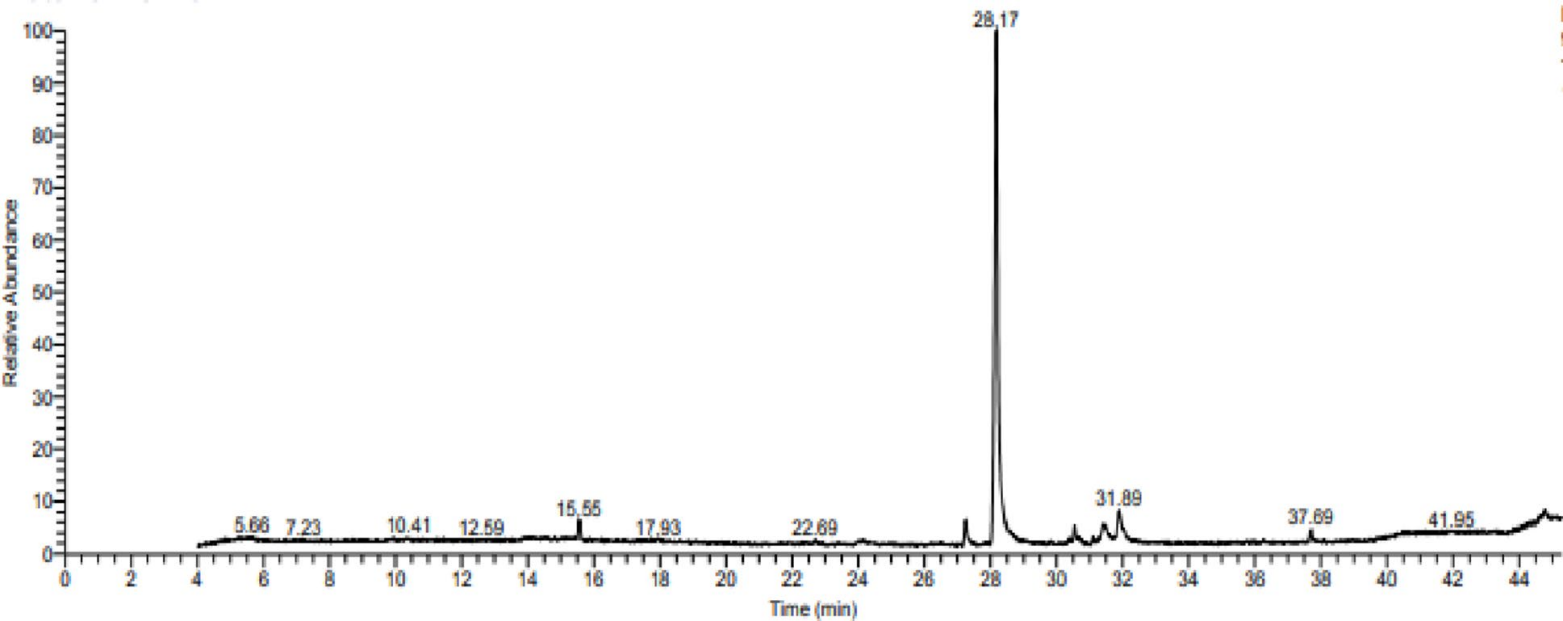
Mass spectrum of biosurfactant produced by *A. xylosoxidans* PX106473

GC-MS analysis of the crude biosurfactant produced by *A. xylosoxidans* PX106473 showed the existence of free fatty acids and fatty acid methyl esters with different carbon chain lengths (Fig. 3). In the chromatogram, a significant peak of hexadecanoic acid or palmitic acid (C_16_H_32_O_2_) occurred at the retention time of 28.17 min. So, hexadecanoic acid was the dominant component with the highest area percentage of 76.44. The other components of extracted biosurfactant include: fatty acids such as octadecanoic acid (stearic acid, C_18_H_36_O_2_) and (9)-octadecenoic acid (oleic acid, C_18_H_34_O_2_). The fatty acid methyl esters identified in the tested biosurfactant include: Hexadecanoic acid methyl ester and 9-octadecenoic acid methyl ester. Moreover, caryophyllene, octadecatrienoic acid (2-Monolinolenin), and Mono (2-ethylhexyl) phthalate were also detected.

### Antibacterial activity of the extracted biosurfactant

**Fig. 4:**
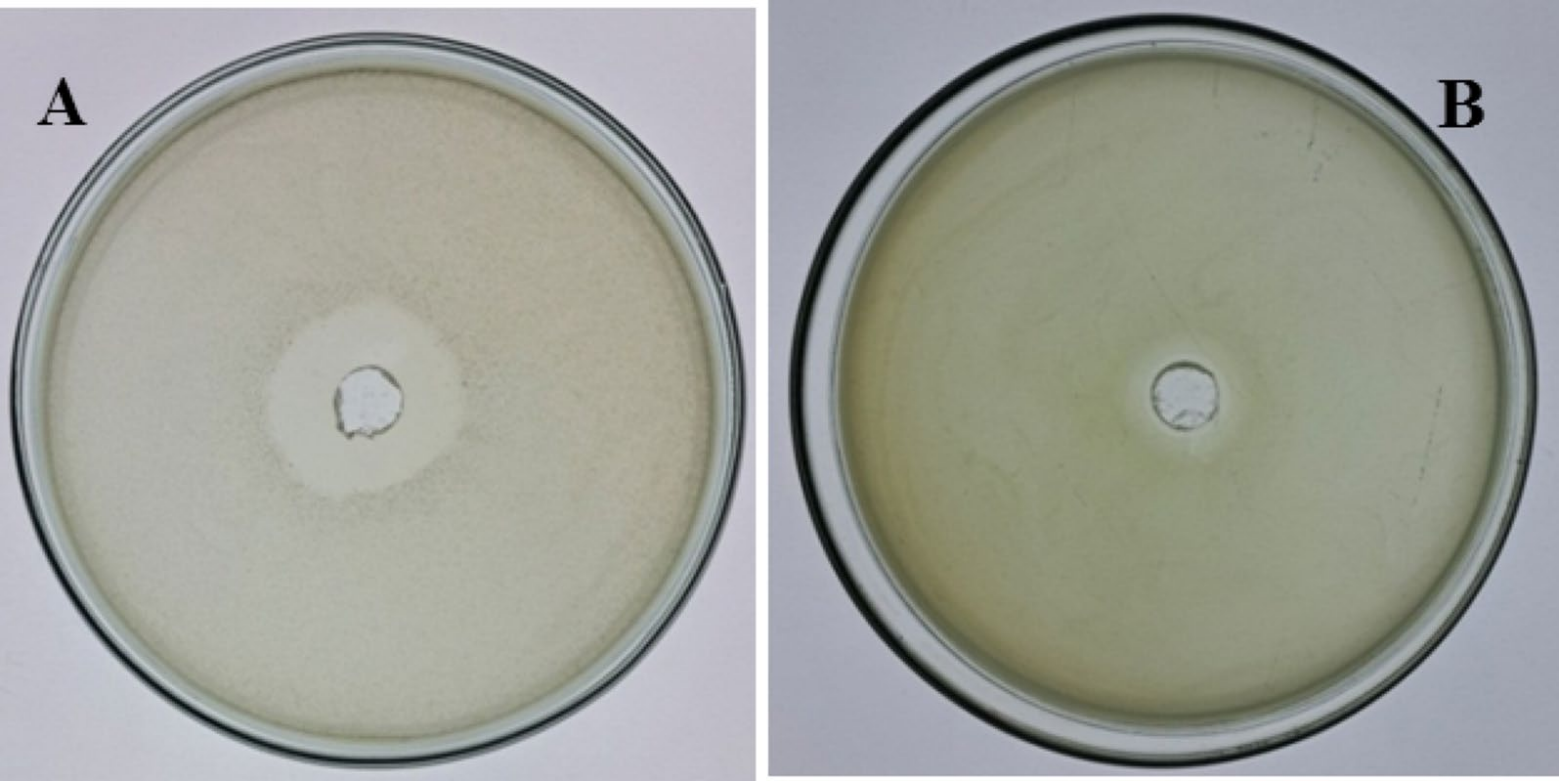
The antimicrobial activity of the biosurfactant extracted from *A. xylosoxidans* PX106473 against *E. coli*, **A** and *S. aureus*, **B**

The antibacterial activity of the biosurfactant extracted from *A. xylosoxidans* PX106473 (190 µg /mL) against representative Gram-negative and Gram-positive strains, *E. coli* and *S. aureus*, was shown in Fig. 4. Results revealed a strong inhibitory activity of the extracted biosurfactant with a clear zone diameter of 26 ± 0.10 mm against *E. coli*, and a notable clear zone against *S. aureus* with 15 ± 0.06 mm diameter (mean ± SD, *n* = 3).

### Docking results of hexadecanoic acid with penicillin-binding protein 2a (PBP2a – 1VQQ) and DNA gyrase subunit B (5MMN)

**Fig. 5:**
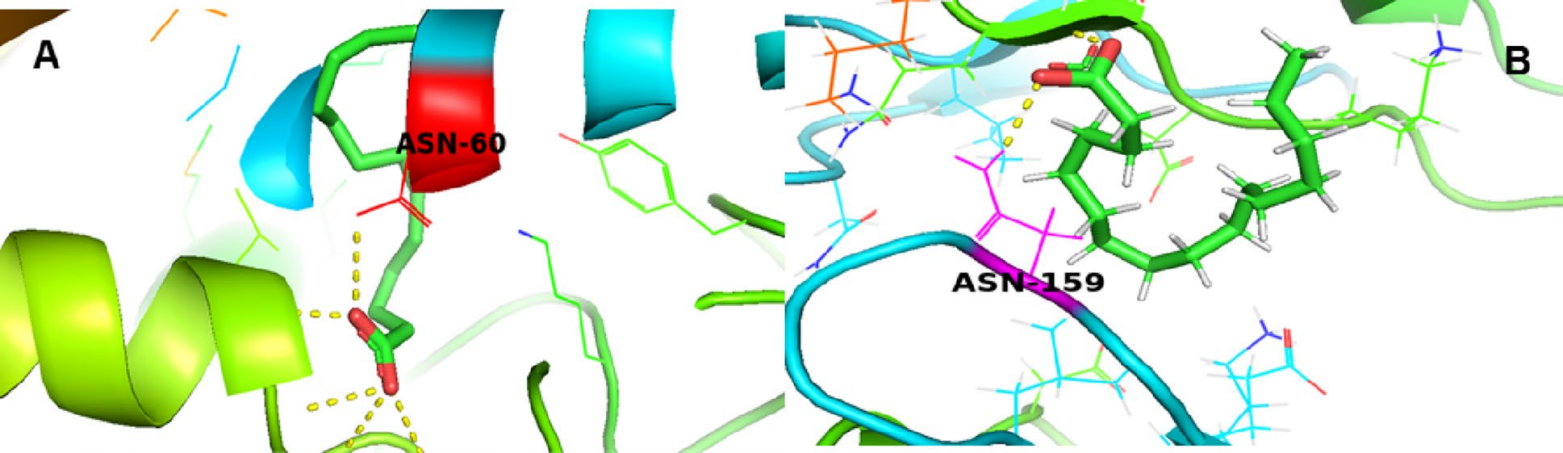
Molecular docking visualization of PBP2a (PDB ID: 1VQQ) with hexadecanoic acid. **A**, and DNA gyrase subunit B (PDB ID: 5MMN) with hexadecanoic acid **B**

Molecular docking studies were performed to assess the binding affinities of hexadecanoic acid phytochemicals against two bacterial target proteins: PBP2a (PDB ID: 1VQQ) and DNA Gyrase Subunit B (PDB ID: 5MMN). The results revealed that hexadecanoic acid displayed potential antibacterial activity based on the docking scores. Hexadecanoic acid showed a notable binding affinity toward the active site of both target proteins. As illustrated in Fig. 5, hexadecanoic acid interacted with PBP2a asparagine at position 159 and formed key interactions within the DNA gyrase active site, specifically with the asparagine residue at position 60. The interactions are likely mediated by hydrogen bonds and hydrophobic forces, which may play a crucial role in impairing the functional activity of DNA gyrase subunit B and contribute to the inhibition of PBP2a function. The docking scores (binding affinities in kcal/mol) of hexadecanoic acid against PBP2a and DNA gyrase subunit B are − 3.9 and − 6.4 kcal/mol, respectively. Lower binding energy values indicate stronger binding affinity.

## Discussion

In this work, a biosurfactant produced by *A. xylosoxidans* PX106473 was isolated and characterized using a low-cost substrate to evaluate its potential for antimicrobial applications. Previous studies had described *A. xylosoxidans* as an effective oil-degrading microorganism and a biosurfactant producer [29, 36, 37]. Various researchers used economical substrates, such as orange peels and waste frying oil, to produce lipopeptide biosurfactants by *Bacillus licheniformis* and *Bacillus* sp. HIP3 [10, 38]. We substituted refined substrates with waste frying oil, a nearly zero-cost and abundantly available material. This substitution reduced the medium cost.

To assess the emulsifying activity (E24%) of biosurfactants, kerosene has commonly been utilized as a standard hydrocarbon in previous studies. As kerosene has been emulsified up to 87% by lipopeptides [39] and 74% by rhamnolipids [18]. In this study, the E24% of the extracted biosurfactant by *A. xylosoxidans* PX106473 was 66.7%, suggesting that it possesses a favorable emulsifying activity. The oil spreading method is quick and simple for detecting biosurfactants present in low concentrations or with low activity [25]. Our results indicated that just 5 µL of fermentation broth produced a large oil displacement zone of 7 cm in diameter, confirming high biosurfactant activity. In certain studies, the blood agar lysis method serves as the only method used in the screening process for biosurfactant production [25]. According to the test principle, biosurfactants can lyse erythrocytes, producing clear zones on blood agar [40]. When *A. xylosoxidans* PX106473 was spread on blood agar plates, the results showed colonies with clear hemolytic circles, indicating that *A. xylosoxidans* PX106473 can produce biosurfactant.

TLC results showed that the extracted biosurfactant by *A. xylosoxidans* PX106473 could be a lipopeptide as it contained both amino acids and lipid groups. From the FT-IR analysis of the tested biosurfactant, a significant broad peak around 3215 cm^–1^ suggests the presence of amides (-NH) groups, which are common in lipopeptides. The presence of C-H stretching vibrations indicates long chains of hydrocarbon. The carbonyl (C = O) functional group strongly supports the presence of ester or carboxyl functional groups, which are critical in biosurfactant structures. Also, the sharp absorption peak corresponding to CO-N bending of amide groups around 1529 cm^-1^ indicates the presence of amino acid groups. These results were similar to the lipopeptide biosurfactants FTIR analysis reported in previous studies [41–43]. Ramani, Jain [41] also noted that the characteristic stretching frequency of amide groups is only present in lipopeptide-type biosurfactants and is generally not observed in glycolipids. These findings suggest that the biosurfactant structure consists of a long hydrocarbon chain conjugated with a peptide moiety, aligning with the findings obtained from the previously described TLC analysis. The composition of the fatty acids in the extracted biosurfactant was analyzed by GC-MS to further reveal its molecular structure. Hexadecanoic acid was found to be the dominant component with 76.44% area percentage according to GC-MS results. Additionally, a lipid-based biosurfactant was suggested as the biosurfactant consisted of various types of long-chain fatty acids, such as hexadecanoic acid, octadecanoic acid, and (9)-octadecenoic acid. Hexadecanoic acid was also the dominant component in the previous study of [44]. The fatty acid composition is not only influenced by the producing bacterial strain but also by the used substrate and the culture conditions [41, 45, 46].

The produced lipopeptide-biosurfactant showed antibacterial activity towards both Gram-negative and Gram-positive bacteria. Peptide-based biosurfactants with antimicrobial properties are often reported [47–51]. To evaluate the antibacterial potential of hexadecanoic acid, the main component of the produced biosurfactant, molecular docking was performed. The analysis revealed that hexadecanoic acid bound to two key bacterial proteins: PBP2a with a binding affinity of -3.9 kcal/mol and DNA gyrase subunit B with − 6.4 kcal/mol. Reference antibiotics, fluoroquinolones such as ciprofloxacin, typically exhibit docking energies of -5.5 to -7 kcal/mol toward GyrB, whereas β-lactams targeting PBP2a show − 6 to -8 kcal/mol [52–54]. These values suggest that hexadecanoic acid has a good affinity for GyrB comparable to fluoroquinolones, but a weaker interaction with PBP2a relative to β-lactams. Docking against GyrB revealed strong binding by hexadecanoic acid, with Asn60 consistently involved in stabilizing ligand interactions across the tested compound. Although not previously identified as a primary binding hotspot, Asn60 is positioned near the ATP-binding site, a critical region for enzymatic function [55]. Prior studies have underscored the importance of residues in this region, supporting its relevance in ligand binding and inhibition [56, 57]. The antibacterial action of hexadecanoic acid has been well documented. Its mode of action primarily involves membrane disruption [58]. Furthermore, hexadecanoic acid can induce oxidative stress, inhibit enzymatic activities, and interfere with signal transduction pathways, collectively impairing bacterial survival [59]. Overall, hexadecanoic acid emerges as a promising dual-target antibacterial agent with predicted inhibitory activity against proteins integral to cell wall synthesis and DNA replication. Therefore, the putative lipopeptide-biosurfactant produced by *A. xylosoxidans* PX106473 could be a promising candidate for biotechnological and biomedical applications.

## Conclusion

In this study, we demonstrated a novel and cost-effective approach for biosurfactant production by employing *A. xylosoxidans* PX106473 and waste frying oil as a renewable substrate. This work introduces new concepts by combining low-cost bioprocessing with structural characterization and molecular docking analysis to explain the antibacterial mechanism of the dominant biosurfactant component. The integration of experimental and in silico approaches provides a deeper mechanistic understanding that has not been previously reported for *Achromobacter*-derived biosurfactants.

The findings revealed that the PX106473 strain efficiently produced a putative lipopeptide biosurfactant exhibiting strong emulsifying activity, high oil displacement potential, hemolytic activity, and notable antibacterial effects against both *E. coli* and *S. aureus*. Characterization through TLC, FT-IR, and GC-MS confirmed the presence of lipid and peptide functional groups, with hexadecanoic acid identified as the major constituent. The observed antimicrobial activity was supported by molecular docking results, which demonstrated stable binding of hexadecanoic acid to DNA gyrase B and PBP2a, suggesting its ability to target essential bacterial pathways.

In conclusion, the biosurfactant produced by *A. xylosoxidans* PX106473 represents a promising, low-cost, and sustainable alternative for environmental and antimicrobial applications. Future studies will focus on the structural elucidation of the biosurfactant using NMR and LC-MS/MS, optimization of fermentation conditions, and on determining its MIC values to better understand its potency. These findings collectively demonstrate the potential of this strain for scalable biosurfactant production and open new avenues for biotechnological and biomedical use.

## Data Availability

All data generated or analyzed during this study are included in this published article.

## Abbreviations

A. xylosoxidans: Achromobacter xylosoxidans
*E. coli*: *Escherichia coli*
*S. aureus*: *Staphylococcus aureus*
E_24_: Emulsification index
TLC: Thin-layer chromatography
FT-IR: Fourier transform infrared spectroscopy
GC-MS: Gas chromatography-mass spectrometry
BHM: Bushnell Hass medium
PBP: penicillin-binding protein
PDB: Protein data bank
